# Finite-discrete element numerical modeling on shield tunnel face stability undercrossing existing pipeline considering discontinuous contact

**DOI:** 10.1038/s41598-025-18212-6

**Published:** 2025-10-03

**Authors:** Yingnan Liu, Huayang Lei, Hongwei Huang, Qinghan Li

**Affiliations:** 1 Department of Geotechnical Engineering, Tongji University, Shanghai, 200092 China; 2 School of Civil Engineering, Tianjin University, Tianjin, 300350 China

**Keywords:** Tunnel face, Instability evolution, Existing pipeline, Soil-pipeline interaction, Finite-discrete numerical simulations, Engineering, Solid Earth sciences

## Abstract

Tunnel face stability is critical for excavation safety when undercrossing a pipeline. Previous studies inadequately address soil-pipeline discontinuous contact effects. This study elucidates instability mechanisms using coupled FDM-DEM simulations calibrated by physical tests. Results reveal pipeline-induced sheltering effects on failure modes, with sheltered zones transitioning from inverted triangular to spindle-shaped as *h*/*D* decreases from 0.5 to 0.1. Evolution of contact force chains show reduced forces ahead of tunnel face and beneath the pipeline drive instability. Surface settlement develops dual troughs flanking the pipeline as *δ*/*D* increases from 0.86 to 6.52%. Limit support pressure demarcates three stages at 0.9*σ*_T0_ and 0.2*σ*_T0_. Crucially, discontinuous contact governs deformation: when ground deformation exceeds pipeline displacement, void-induced separation triggers stress loosening. Parametric studies confirm vertical spacing and flexural stiffness influence pipeline settlements. Controlling face support pressure to prevent excessive discontinuous contact is essential for pipeline safety, providing insights for urban tunnelling adjacent to existing structures.

## Introduction

With the development of economic and urbanization in China, an increasing number of tunnels are being constructed to meet growing transportation demands. Maintaining the stability during excavation is particularly critical for construction safety^1–4^. Inadequate control of face support pressure may lead to water inrush, collapse, and other failures^5^, posing significant risks to personnel, environment, and surrounding buildings. Therefore, clarifying the instability evolution characteristics of shield tunnel construction is pivotal for preventing disaster occurrence. Through model tests and numerical simulations, scholars have captured the instability evolution characteristics under active^6^ and passive failure modes^7,8^, revealing collapse mechanisms in sandy and cohesive soils^9–11^, as well as shallow and deep burial conditions^12^. Further investigations have explored failure modes under diverse geological environments, including layered ground^13,14^, seepage flow^15^, and unsaturated ground conditions^16–18^. These studies elucidate the instability evolution mechanisms and limit support pressure during single-tunnel construction, providing valuable insights for tunnel design and construction.

With the rapid development of urban underground space, the construction of shield tunnel adjacent to existing underground structures has become increasingly common, in which the construction-induced ground loss triggers additional deformation of existing underground structures. Previous studies primarily employ field monitoring^19–23^, model tests^24–27^, numerical simulations^28–31^, and theoretical analyses^28,32–35^ to investigate the environmental responses induced by adjacent shield tunnelling, addressing the key factors affecting the deformation of existing underground structures. Both experimental and numerical simulation methods are considered proven methodologies for investigating the interaction effects during adjacent shield tunnelling. Zheng et al^26^ used 1 g physical model tests to analyze how twin-tunnel spacing and volume loss affect surface settlement patterns in granular soils. Lei et al^27^ revealed ground settlement behavior by 1 g model tests during overlapped tunnelling and established a predictive model for deformation of existing tunnel. Lei et al^28^ proposed a phased mechanical model for shield tunnel face instability considering soil arch evolution. Lin et al^29^ employed numerical simulation validated by field data to investigate the response of existing tunnels to obliquely undercrossing twin shield tunnelling. Islam and Iskander^30^ conducted 3D finite element analysis to examine settlement interactions during twin-tunnel excavation considering variations in cover depth, pillar width, and construction sequence. Nevertheless, these studies mainly focus on the deformation responses induced by shield tunnelling disturbances under conventional construction conditions. The stability of shield tunnel face under adjacent construction scenarios is of greater significance, as instability not only leads to the failure of the newly constructed tunnel but also influence the operational safety of the adjacent existing underground structures, potentially triggering severe regional hazards.

Up to now, progress has been achieved on the tunnel face stability by considering adjacent existing underground structures(s), for example, the ground deformation and failure mechanisms during shield undercrossing an existing tunnel were clarified by using numerical simulations^36–38^. Transparent soil model tests conducted by Lei et al.^5^ revealed the actual instability modes of tunnel face under similar conditions. Liu et al.^39^ established a three-dimensional stability model to derive the limit support pressure for tunnel face, considering soil properties, tunnel spacings, and construction stages. Shi et al.^40^ conducted model test of shield undercrossing existing pipeline, investigating deformation behavior of pipeline in response to shield face instability and providing corresponding calculation charts. However, these studies predominantly address soil stability subjected to adjacent shield tunnelling, neglecting the responses of existing underground structures to variations in face support pressure. This oversight is particularly critical for flexible structures like existing pipeline, which is more susceptible to excessive deformation when subjected to instability of adjacent shield tunnel face instability. Incompatibility between soil deformation induced by face instability and pipeline deformation may trigger void formation at the soil-pipeline interface. Concurrently, the degradation of existing pipeline performance further destabilizes the new tunnel face, creating a coupling effect that accelerates the instability evolution.

Numerical methods provide robust tools to address these interaction challenges. While the discrete element method (DEM), capable of simulating large soil deformations, has been widely applied to tunnel face stability analysis^41–44^. However, modeling existing pipeline as structural elements using DEM remains problematic even if it can be approximated by stacking and combining a series of particles using parallel bond contact model^45^. The inherent differences in mechanical behavior between discontinuous granular assemblies and continuum structures complicate parameter calibration. A viable solution lies in employing continuous structural elements to simulate existing pipeline, effectively bridging the gap between discrete and continuum modeling approaches. The coupled finite-discrete element method is a well-established numerical technique since its pioneering development by Munjiza et al.^46^ in the final decade of the twentieth century. It synergizes the strengths of analyzing continuum mechanics problems with the capability of handling non-continuum, large displacements, and contact interactions^47,48^. Recently, Li et al.^49^ proposed a grout diffusion model in loess tunnels using a coupled finite-discrete element method, providing valuable references for the design and construction of shield tunnels in loess strata. Li and Liu^50^ introduced a novel combined finite-discrete element method to analyze spatial effects during tunnel excavation. This demonstrates that employing coupled finite-discrete element method is highly suitable for analyzing the mechanical responses of surrounding soil and existing structure induced by tunnel excavation.

This study investigates the mechanical response of surrounding ground and existing pipeline induced by face instability during shield undercrossing. By integrating discrete particles for modeling soil and continuum elements for modeling existing pipeline, the interactive mechanisms between the shield tunnel face, ground, and existing pipeline are clarified. The catastrophic evolution process—ranging from initial face instability, failure propagation, structural degradation, to further instability—is systematically revealed. This paper enhances the understanding of failure mechanisms in adjacent tunnelling and contributes to the construction safety of urban underground infrastructure.

## Finite-discrete numerical modelling

### Software and principles

Finite difference method (FDM) can primely simulate tunnel structure and has high computational efficiency. Discrete element method (DEM) can effectively reproduce the micro mechanical behavior of soil particles^51^ and reveal the instability mechanism of shield tunnel face adjacent to existing pipeline. Hence, the interaction of shield-ground-pipeline can be effectively analyzed by using coupled FDM-DEM numerical simulation. FLAC^3D^ software^52^ and PFC^3D^ software^53^, which are based on FDM and DEM, respectively, were used to simulate the response of ground and existing pipeline to face instability of an undercrossing shield tunnel. FLAC^3D^ provides an interface for coupling with PFC^3D^, enabling both software packages to be loaded into the same user interface and utilized simultaneously. This allows continuum and discrete element models to coexist within a single modeling framework. The interaction between FLAC^3D^ and PFC^3D^ requires coupling modules, there are three coupling schemes:

1. 1D Structural Element Coupling, which couples PFC^3D^ components to 1D structural elements in FLAC^3D^.
2. Wall-Zone Coupling, which couples PFC^3D^ components to shell-based structural elements and/or zone faces in FLAC^3D^.
3. Ball-Zone Coupling, which couples PFC^3D^ ball elements to zones in FLAC^3D^.

Pipeline is typically modeled using structural elements, so that the Wall-Zone Coupling scheme is adopted in this study. Walls in PFC^3D^ are aligned with the surfaces of lining elements in FLAC^3D^, composed of edge-connected triangular facets. The vertices of these facets are assigned velocity and position as functions of time. The coupling mechanism operates as follows: firstly, the contact forces and moments between PFC^3D^ particles and walls are retrieved. Then, the equivalent force systems are determined at facet vertices. Finally, the forces and stiffness contributions are transferred to FLAC^3D^ mesh nodes.

**Fig. 1:**
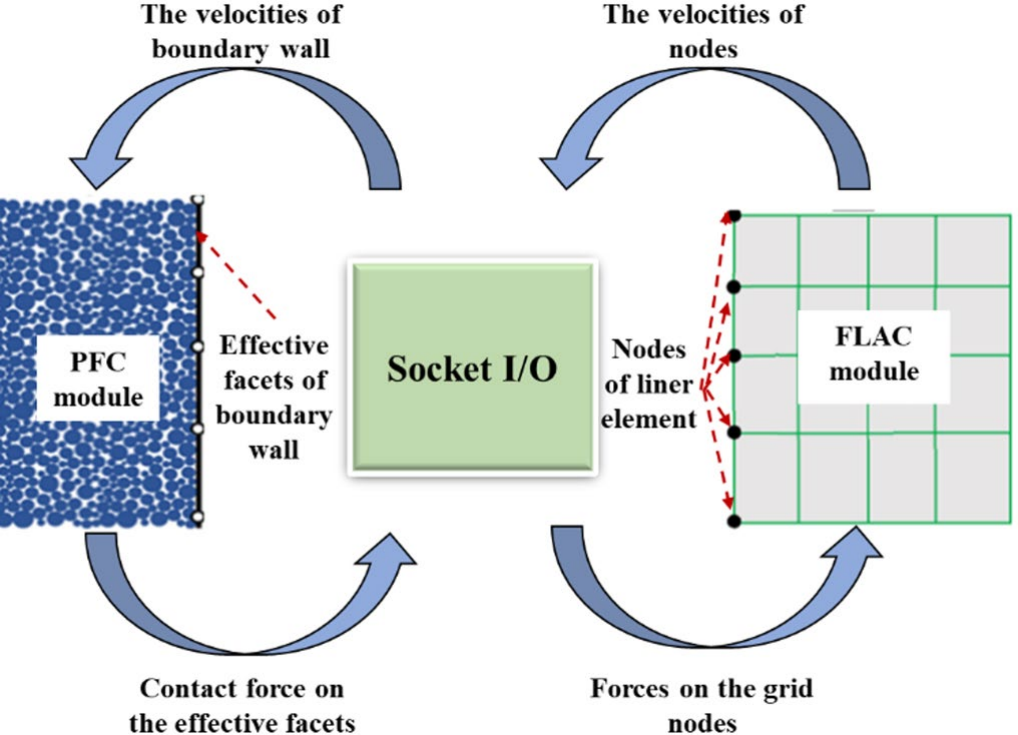
Coupling mechanism between PFC^3D^ and FLAC^3D^.

This coupling method bridges macroscopic and microscopic mechanics, enabling simulations of shield tunnel face instability mechanisms (e.g., particle migration and failure in surrounding ground) and their interactions with existing pipeline. Such simulations holistically capture microstructural evolution and macro-mechanical responses. As illustrated in Fig. 1, data exchange between PFC^3D^ and FLAC^3D^ is achieved via Socket I/O function, an inter-process communication protocol. At each computational timestep, PFC^3D^ boundaries are defined by articulated effective facets that conform to mesh deformations in FLAC^3D^. Nodes of these facets share identical coordinates with FLAC^3D^ mesh nodes, ensuring synchronized motion. Node velocities from FLAC^3D^ (under large deformations) are transferred to PFC^3D^, enabling effective facets to move identically to FLAC^3D^ meshes. Forces generated by particle–wall interactions in PFC^3D^ are transmitted back to FLAC^3D^ mesh nodes. This iterative data loop at each timestep accurately replicates soil-structure interactions, making it particularly effective for studying bidirectional coupling effects between tunnel face, ground, and existing pipeline.

### Establishment of numerical model

In order to accurately simulate the instability evolution of shield tunnel face adjacent to existing pipeline, the soil and tunnel face were simulated by balls and wall in PFC^3D^, respectively, and the existing pipeline was simulated by liner element in FLAC^3D^.

The physical and mechanical parameters of soil were based on the sand in the model tests conducted by Shi et al.^40^, the sand has a mean diameter *D*_50_ of 0.8 mm and a friction angle *φ*_*c*_ of 30.7°. The diameters of the tunnel (*D*) and pipeline (*D*_0_) are 230 mm and 20 mm, respectively. Since the time consumption increases exponentially with the particle number, it is not possible to establish model according to the particle diameter of soil in reality. Previous research^54^ has proved that the method of increasing particle radius to reduce computational complexity has limited impact on simulation results as long as the ratio of tunnel diameter to mean particle diameter *D*/*D*_50_ is larger than 12. Therefore, in order to improve simulation efficiency, the soil particle diameter within a radial distance of 6*D*_0_ from the pipeline axis is doubled. (*D*_50_ = 1.6 mm, namely, *D*/*D*_50_ = 143.75), while the particle size in other areas is enlarged by 8 times (*D*_50_ = 6.4 mm, namely, *D*/*D*_50_ = 35.94). Although the scaled soil particle size (1.6 mm) exceeds the pipeline thickness, it remains significantly smaller than the pipeline diameter (20 mm), ensuring uniform stress distribution. In addition, according to Deng et al^55^, when the Young’s modulus of a particle is constant, the stiffness of the particle is related to its radius as follows:

1 $$\overline{E} = \frac{{k_{n} }}{4R}$$

where $\overline{E}$ is the Young’s modulus of the particle, N/m^2^; *k*_*n*_ is the stiffness of the particles, N/m; *R* is the average particle size of the particles, m.

**Fig. 2:**
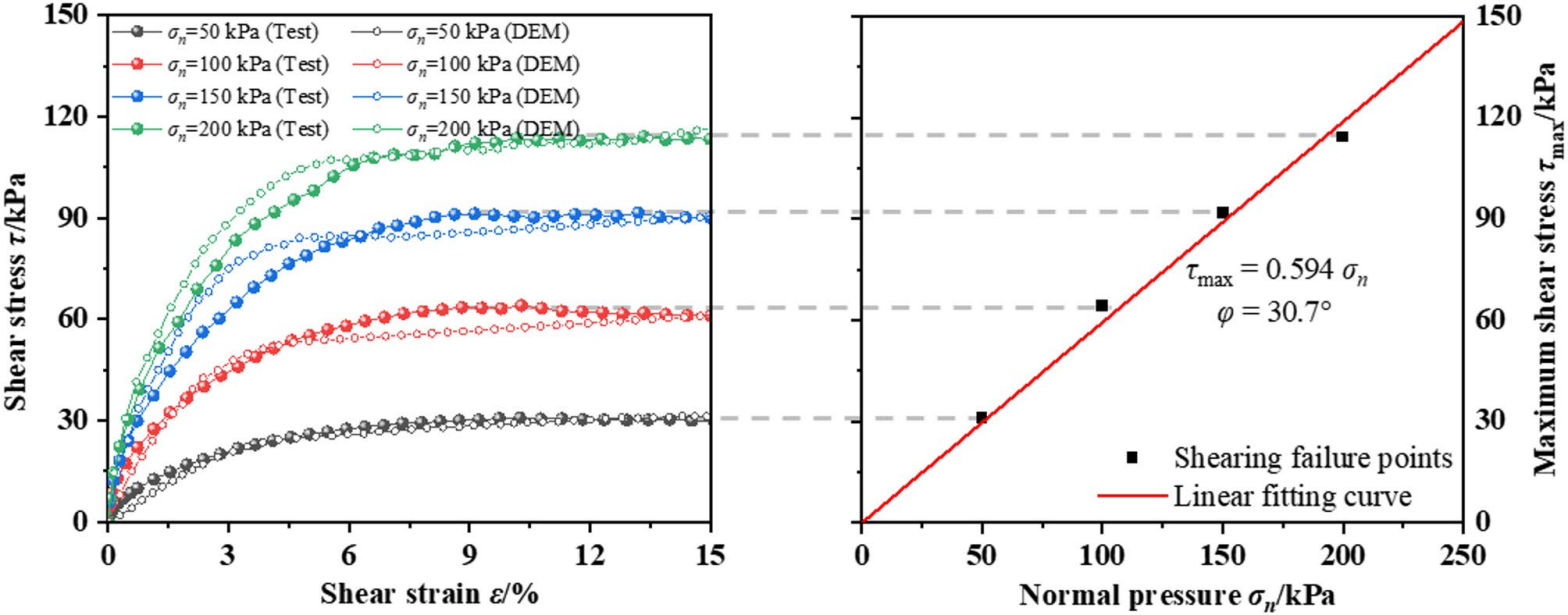
Friction angle obtained by direct shear test from test^40^ and DEM simulation.

Due to the lack of a one-to-one correspondence between the microscopic and macroscopic mechanical parameters of particles in the DEM, it is necessary to calibrate the microscopic parameters. The soil parameters were calibrated using direct shear test. As the soil belongs to sand, the linear model was adopted, consisting of normal stiffness *k*_n_, tangential stiffness *k*_s_, and friction coefficient *μ*. In DEM simulations, linear springs are adopted for the contacts between soil particles to account for normal and tangential interactions, effectively capturing the elastic and frictional interactions between soil particles. The calibration of soil parameters adopts the trial-and-error method, i.e. continuous adjustment of the microscopic parameters until the selected parameters can simulate the stress–strain curve to be consistent with that obtained from test. Figure 2 presents the comparison between experimental results (Shi et al.^40^) and DEM simulations in this study. The minor deviations observed confirm that the DEM parameter selection is realistically representative. The calibrated parameters are listed in Table 1.

**Table 1:** Parameters for soil particle in PFC^3D^.

Mean diameter *D*_50_ (mm)	Specific gravity *G*_*s*_	Void ratio *e*	Density *ρ*_*s*_ (kg·m^−3^)	Normal stiffness *k*_*n*_ (N·m^−1^)	Tangential stiffness *k*_*s*_ (N·m^−1^)	Friction coefficient *f*_*c*_
1.6	2650	0.53	1732	1.0e8	5.0e7	0.6
6.4	2650	0.53	1732	4.0e8	5.0e7	0.6

The existing pipeline was modeled using the liner elements in FLAC^3D^, and the mechanical parameters of polyvinyl chloride (PVC) were adopted for the liner elements as shown in Table 2, which are consistent with those in the model tests conducted by Shi et al.^40^.

**Table 2:** Parameters for existing pipeline in FLAC^3D^.

Pipeline diameter *D*_0_ (mm)	Thickness *t* (mm)	Density *ρ*_0_ (kg·m^−3^)	Elastic modulus *E* GPa	Poisson’s ratio *μ*
20	1.4	2500	1.0	0.2

**Fig. 3:**
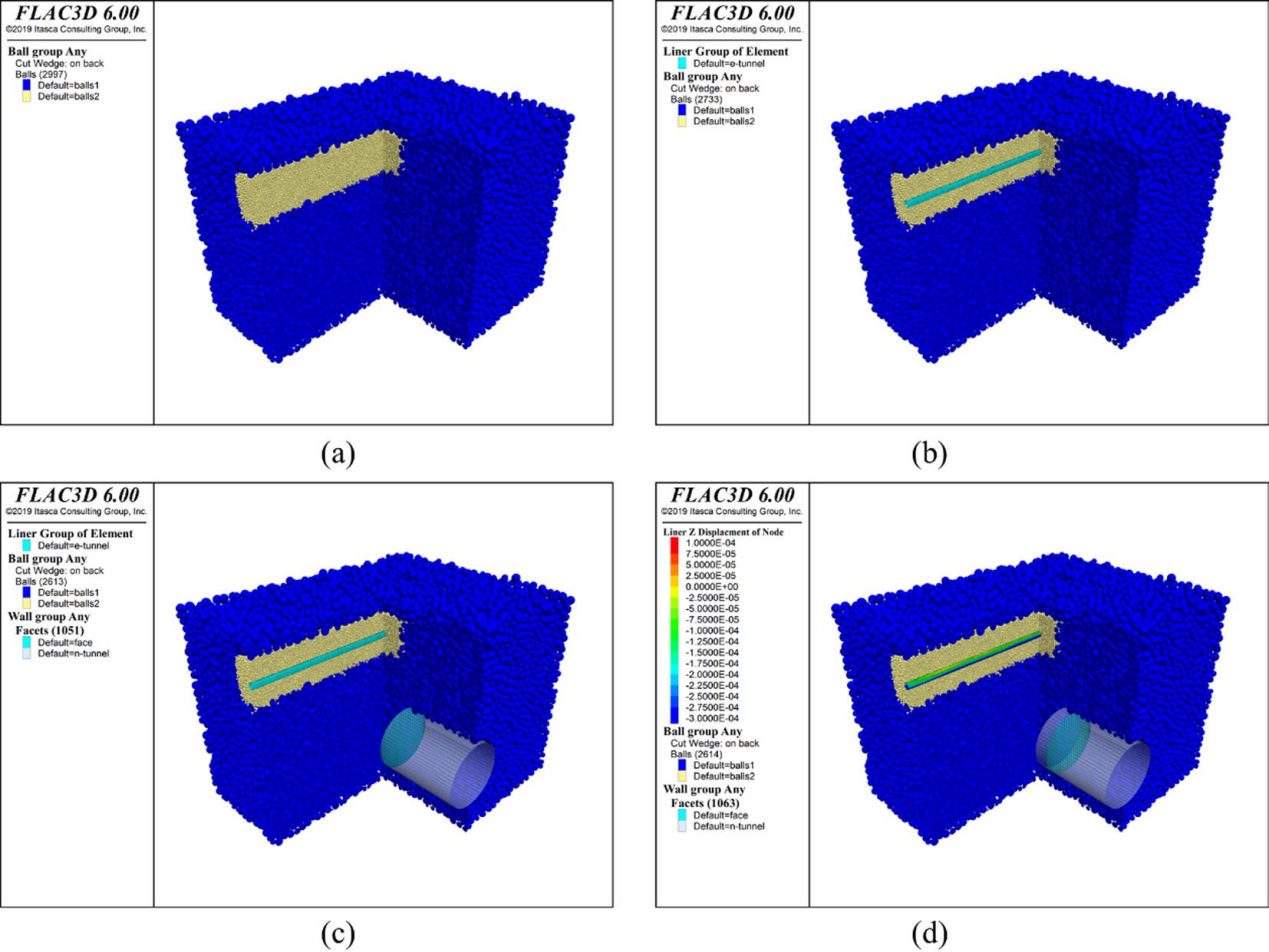
Establishment of the numerical model. (**a**) Initial earth pressure balance, (**b**) Generation of the existing pipeline and re-balance, (**c**) Generation of the new tunnel and addition of the tunnel face, (**d**) Simulation of the tunnel face instability by displacement-controlled method (DCM) (Software: FLAC3D 6.00, https://www.itascacg.com/software/flac3d).

After determining the parameters for soil particles and the existing pipeline, the coupled PFC^3D^-FLAC^3D^ numerical model for simulating shield tunnel face instability adjacent to existing pipeline was constructed, as shown in Fig. 3. The process began by generating the foundation soil using the radius enlargement method^5^ and achieving initial equilibrium under initial ground stress (Fig. 3a). It should be noted that the ground was sectioned for visualization purposes, while the actual model remained intact, as similarly depicted in subsequent figures. Next, the existing pipeline was created using liner elements in FLAC^3D^, followed by secondary stress equilibrium to account for its structural presence (Fig. 3b). Subsequently, the new tunnel was excavated, and a support plate was installed at the tunnel face to ensure the tunnel face stability (Fig. 3c). Finally, the instability of the tunnel face was simulated, with continuous monitoring of face support pressure, ground deformation, and responses of the existing pipeline (Fig. 3d).

In the simulation of shield tunnel face instability, stress-controlled method (SCM) or displacement-controlled method (DCM) are commonly employed^9,56–60^. The stress-controlled method typically involves the gradual release of pressure, while the displacement-controlled method generally utilizes the gradual retraction of a rigid plate. The displacement-controlled method performs better in capturing strain softening of soil during tunnel face instability^61^. Therefore, this study adopts the displacement-controlled method that gradually retracting the tunnel face to replicate the face instability.

### Simulation scheme

The purpose of the numerical simulation is to investigate the progressive failure mechanism of the ground and existing pipeline induced by the instability of shield tunnel face from macro and micro perspectives. A total of 12 groups of numerical simulations were conducted, with each simulation replicating tunnel face instability using the DCM (Table 3).

**Table 3:** Simulation schemes.

Case	Cover depth of the new tunnel *C*/*D*	Vertical spacing *p*/*D*	Horizontal distance* h*/*D*	Flexural stiffness* E*_*p*_*I*_*p*_ (N⋅m^2^)
1	1.0	0.5	0	4.16
2	1.0	0.5	0.1	4.16
3	1.0	0.5	0.3	4.16
4	1.0	0.5	0.5	4.16
5	1.5	1.0	0	4.16
6	1.5	1.0	0.1	4.16
7	1.5	1.0	0.3	4.16
8	1.5	1.0	0.5	4.16
9	0.6	0.2	0.3	4.16
10	1.0	0.7	0.3	4.16
11	1.0	0.5	0.3	2.08
12	1.0	0.5	0.3	6.24

Different burial depths (*C*/*D* = 1.0 and 1.5) and excavation distances (i.e., the horizontal distance between the tunnel face and the axis of the existing pipeline, *h*/*D* = 0, 0.1, 0.3, and 0.5) were considered, which are in consistent with Shi et al.^40^. In addition, due to the fact that the vertical spacing and the flexural stiffness are also important factors affecting the mechanical response of the existing pipeline, various tunnel spacings (*p*/*D* = 0.2, 0.5, and 0.7) and flexural stiffness (*E*_*p*_*I*_*p*_ = 2.08N⋅m, 4.16 N⋅m, and 6.24 N⋅m.) were also considered. Underground pipelines were commonly buried at a depth of 1.0 to 3.0 m (e.g., Vorster et al.^33^; Shi et al.^40^). In the model tests, the cover depth of the existing pipeline was designed as 0.5*D* (Case 1–8, 11, 12), 0.4*D* (Case 9), and 0.3D (Case 10), corresponding to 3.0 m, 2.4 m, and 1.8 m in practice. Besides, The Young’s modulus of the PVC tube was 1.0 GPa, giving a flexural stiffness (*E*_*p*_*I*_*p*_) of 4.16 N⋅m^2^. By converting it to prototype scale, the flexural stiffness of the pipeline was 3.4 MN⋅m^2^. In practice, PVC tubes usually a flexural stiffness of 3.2 MN⋅m^2^(e.g., Shi et al.^40^), which indicates that the flexural stiffness of the pipeline in numerical simulation is within the actual range. Throughout the simulations, ground deformation, surface settlement, contact force chains, and the pipeline deformation were monitored to comprehensively assess the impact of tunnel face instability on surrounding ground and existing pipeline.

### Validation of numerical model

**Fig. 4:**
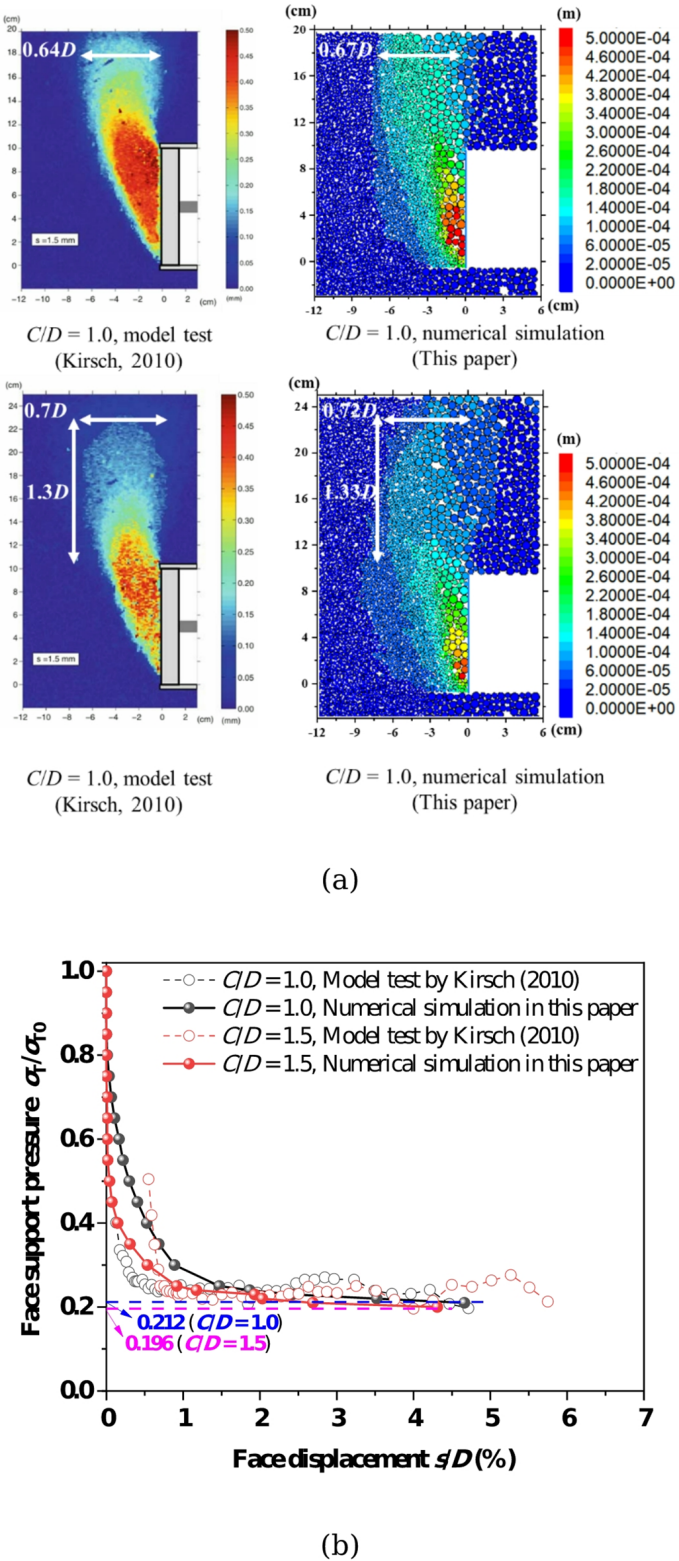
Validation of numerical model by comparison between the model tests results (Kirsch^9^) and this paper. (**a**) Failure modes, (**b**)Support pressure–displacement curves.

The discrete element method (DEM) based numerical simulation employed in this study is first validated for tunnel face stability analysis under single tunnel condition. Based on the model tests conducted by Kirsch^9^, the face failure modes of single tunnel were compared, as illustrated in Fig. 4a. The results indicate that the deformation and failure zones obtained from both model tests and numerical simulations are basically consistent, with discrepancies within 5%. Figure 4b compares the face support pressure–displacement curves derived from model tests and numerical simulations at *C*/*D* = 1.0 and 1.5. The dashed lines represent the corresponding limit support pressures. The curves exhibit consistent trends between model tests and numerical results, and the derived limit support pressures align closely. This agreement validates the capability of the numerical model to investigate tunnel face instability.

**Fig. 5:**
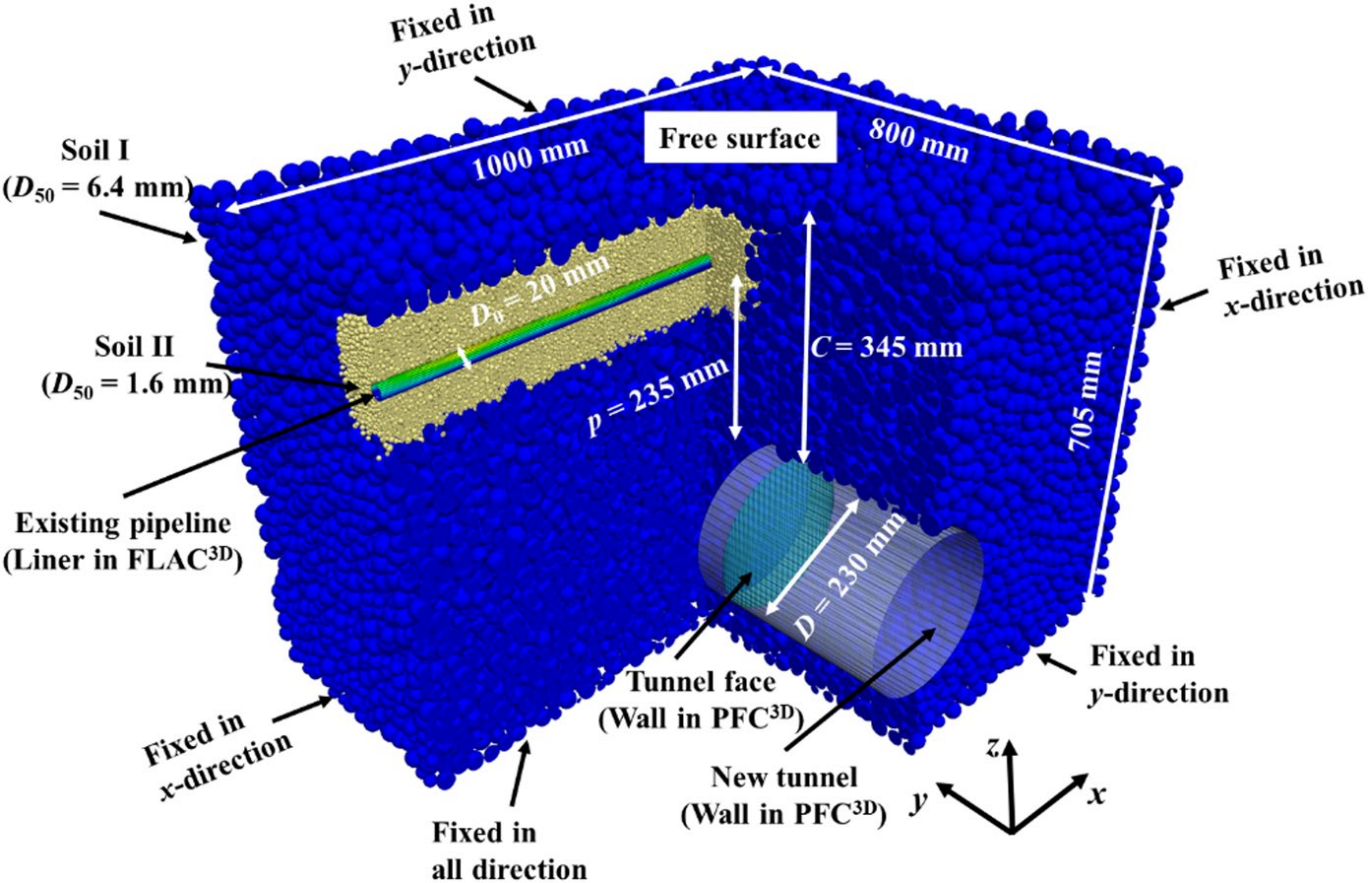
The established finite-discrete numerical model (Software: FLAC3D 6.00, https://www.itascacg.com/software/flac3d).

**Fig. 6:**
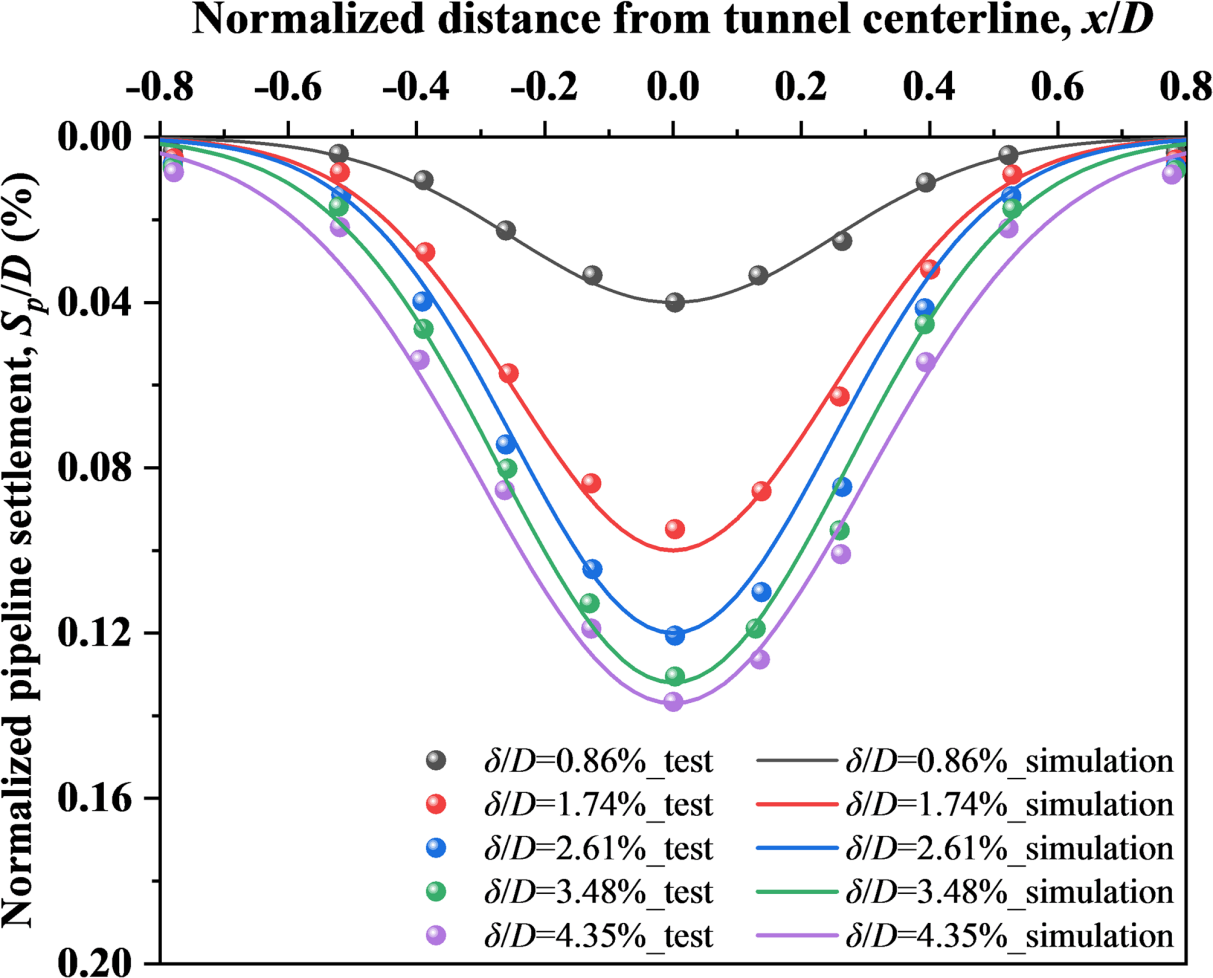
Comparison of pipeline deformation at different tunnel face movements (*δ*/*D*) between model tests (Shi et al.^40^) and numerical simulations.

Subsequently, validation is conducted for the FDM-DEM numerical model of tunnel face stability during shield undercrossing an existing pipeline. The established numerical model is shown in Fig. 5, where the model size and the specific location relationship between the existing pipeline and the tunnel have been indicated. The model boundary conditions are specified as follows: the bottom boundary is fixed, the top boundary is free, and all side boundaries are fixed in the normal direction. Based on the model tests conducted by Shi et al.^40^, the pipeline settlements at different tunnel face movements (*δ*/*D*) were compared, as illustrated in Fig. 6. The results indicate that the pipeline settlement obtained from both model tests and numerical simulations are basically consistent. This agreement validates the capability of the numerical model to investigate tunnel face instability undercrossing an existing pipeline.

## Numerical results

### Ground response to face instability

#### Failure modes under different horizontal distances

**Fig. 7:**
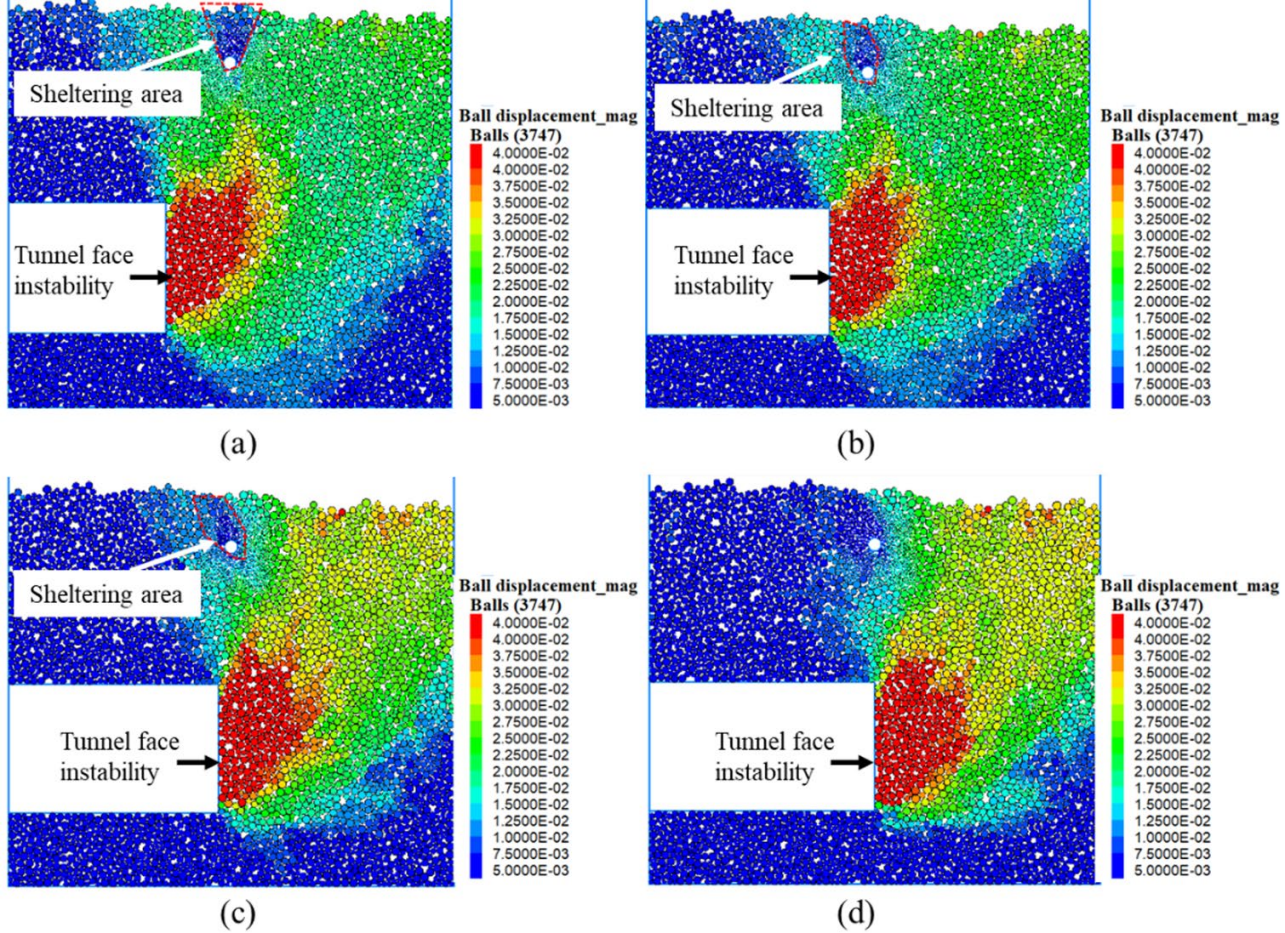
Ground displacement induced by face instability of an undercrossing shield tunnel at different horizontal distances. (**a**) *h*/*D* = 0.5, (**b**) *h*/*D* = 0.3, (**c**) *h*/*D* = 0.1, (**d**) *h*/*D* = 0 (Software: FLAC3D 6.00, https://www.itascacg.com/software/flac3d).

Different horizontal distances between the tunnel face and the axis of the existing pipeline were considered to investigate the influence of tunnel face instability on the ground during shield advancing, as shown in Fig. 7. It can be seen that the ground failure patterns induced by tunnel face instability significantly are affected by the ratio of horizontal distance to diameter (*h*/*D*). The ground failure modes generally show a wedge-shaped region and the major deformation area ranged approximately 1*D* ahead of the tunnel face. Furthermore, observations reveal that when the deformation zone induced by tunnel face instability propagates toward the existing pipeline, the pipeline exhibit a sheltering effect that inhibits further deformation progression toward the ground surface. As the tunnel face progressively approaches the existing pipeline, the deformation region influenced by sheltering effect undergoes significant changes. At *h*/*D* = 0.5, the sheltering area primarily manifests as an inverted triangular zone directly above the pipeline. While at *h*/*D* = 0.3 and 0.1, the dominant affected area shifts to a spindle-shaped zone deflected away from the tunnel face direction above the pipeline. Finally, when *h*/*D* = 0, the sheltering effect essentially vanishes because the existing pipeline lies beyond the influence region of face instability.

#### Microscopic mechanism of face instability

**Fig. 8:**
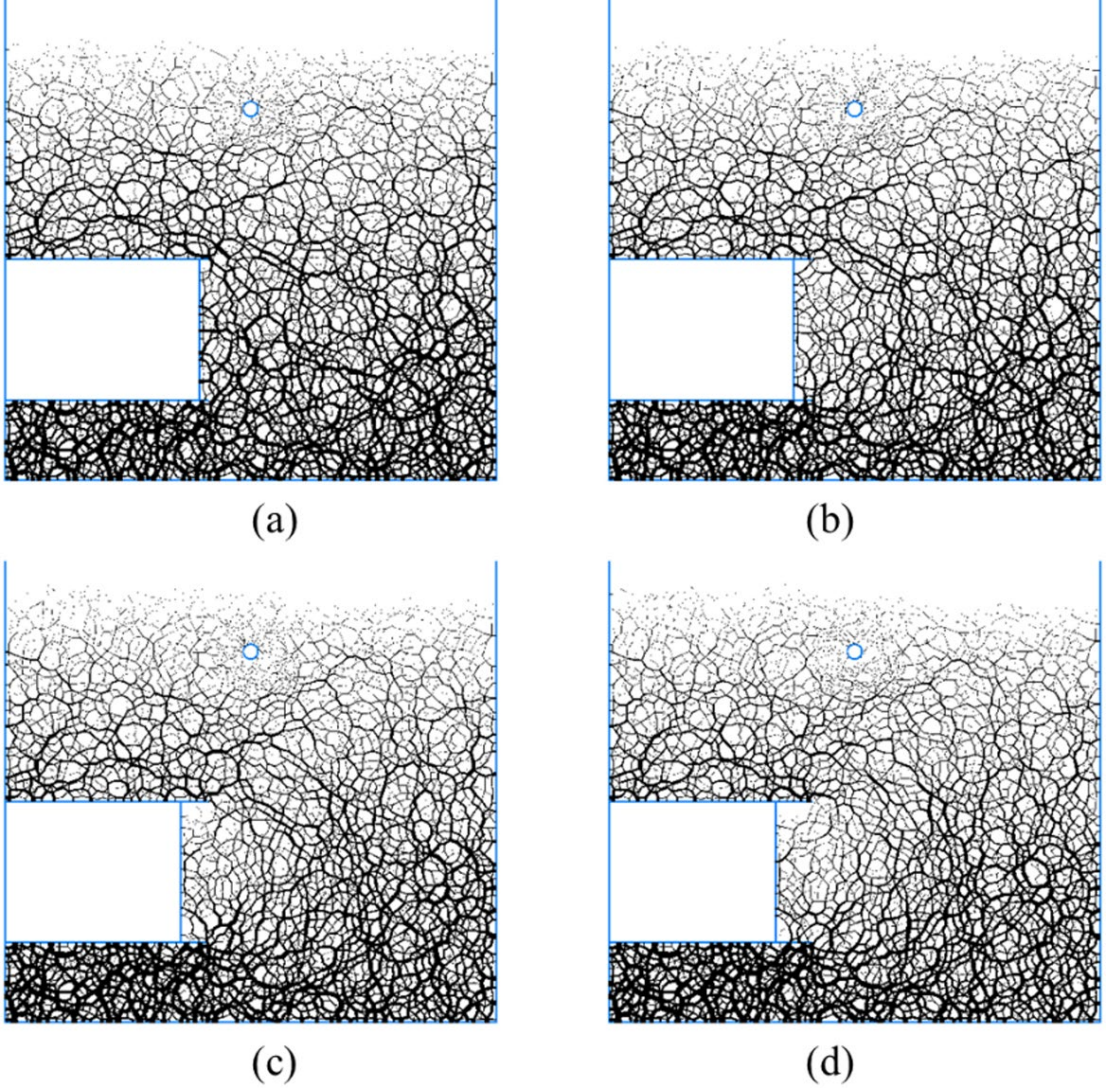
Variations of contact force chain during the instability process. (**a**) *δ*/*D* = 0.86%, (**b**) *δ*/*D* = 3.48%, (**c**) *δ*/*D* = 5.65%, (**d**) *δ*/*D* = 6.52% (Software: FLAC3D 6.00, https://www.itascacg.com/software/flac3d).

The instability of shield tunnel face induces stress redistribution within the ground, which can be effectively characterized through the contact force chain of particles in PFC^3D^ as illustrated in Fig. 8, which shows the variations of contact force chain during the instability process of the tunnel face.

The support pressure is transmitted through the tunnel face to the nearby soil, and then further transmitted to the ground through the contact of soil particles. Therefore, when the support pressure is within an appropriate range, a balanced state of tunnel face can be maintained. Once the support pressure is insufficient to maintain the balance state, the slip and migration between soil particles continuously generate new contact force chains. The contact force chains of the soil below the existing pipeline becomes sparse as it blocks the path of the transmission of force chain, which is the micro reason for the instability of the tunnel face undercrossing the existing pipeline.

#### Ground surface settlement development

**Fig. 9:**
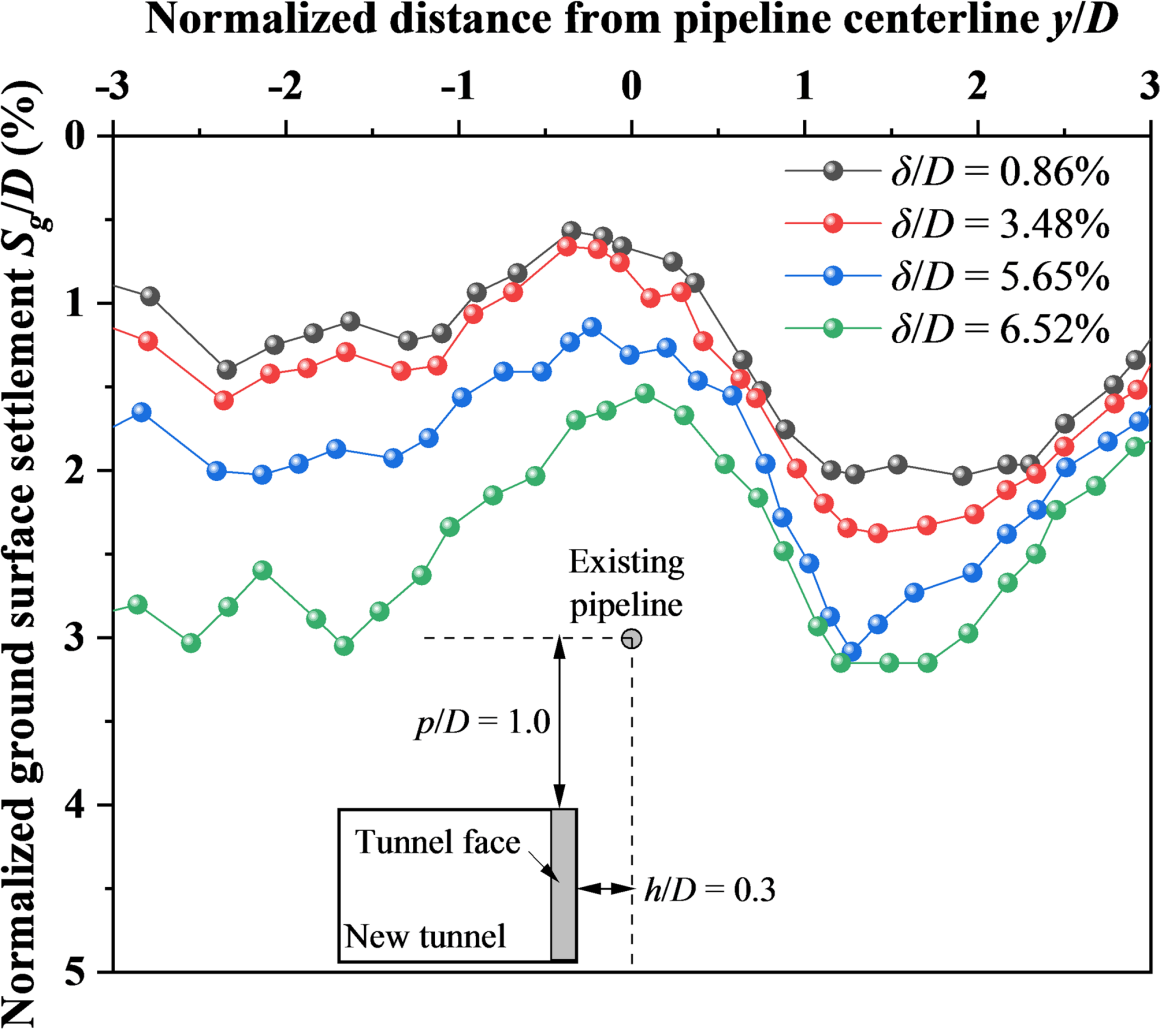
Ground surface settlement at different tunnel face movements.

Figure 9 exhibits the ground surface settlement along the longitudinal section of shield tunnel at different tunnel face movements when *p*/*D* = 1.0 and *h*/*D* = 0.3. The results clearly reveal the formation of settlement troughs on both sides of the existing pipeline. As the tunnel face movement increases, the depth of these troughs intensifies. When the tunnel face movement reaches *δ*/*D* = 6.52%, the settlements on both sides exceed 3%*D* (6.9 mm), indicating significant ground loss around the existing pipeline. This observation underscores the critical relationship between tunnel face movement, soil mass redistribution, and structural vulnerability during shield tunnelling.

In summary, the DEM simulations have established a mechanistic link between the microscopic force chain evolution and the macroscopic deformation patterns. As the tunnel face approaches the existing pipeline, the pipeline obstructs force chain transmission, inducing localized sparseness of contact forces beneath it (Fig. 8). This disruption weakens the overlying soil arch, triggering stress redistribution along the shear bands that transform the initial inverted triangular deformation zone into a deflected spindle-shaped zone above the pipeline (Fig. 7). Concurrently, these redirected shear bands propagate as conjugate slip surfaces toward the ground, forming a V-shaped sliding channel. Soil blocks between the shear bands slide downward along this channel, collectively generating asymmetric dual troughs flanking the pipeline (Fig. 9).

### Soil-pipeline discontinuous contact mechanisms

**Fig. 10:**
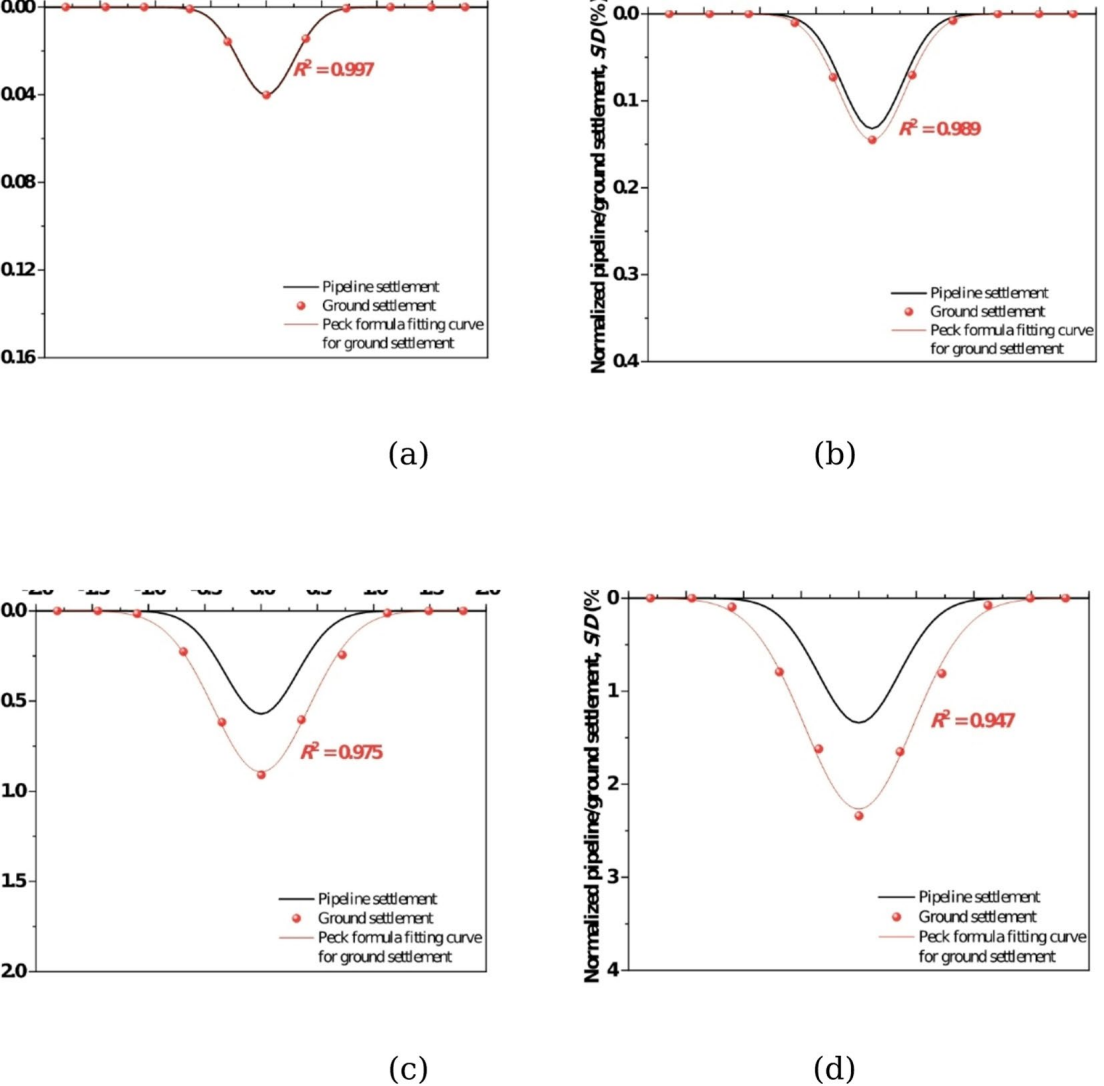
Settlement of ground and existing pipeline at different tunnel face movements. (**a**) *δ*/*D* = 0.86%, (**b**) *δ*/*D* = 3.48%, (**c**) *δ*/*D* = 5.65%, (**d**) *δ*/*D* = 6.52%.

Figure 10 shows the deformation of ground and existing pipeline at different tunnel face movements. It can be seen that when the tunnel face movement *δ*/*D* = 0.86%, the ground and the existing pipeline are in a continuous contact state, with a maximum settlement about 0.04*D*% (0.1 mm). As the tunnel face movement *δ*/*D* reaches 3.48%, a void zone appears between the ground and the existing pipeline. The emergence of the void zone signifies a transition in the pipe-soil interaction induced by instability of the undercrossing shield tunnel face: from continuous contact at small deformation to discontinuous contact at large deformation. With the further movement of the tunnel face, the void zone becomes larger and larger, when tunnel face movement is 6.52%, the differential settlement (i.e., the height of the void zone) reaches 2.3 mm (74.8 mm in prototype), indicating that the instability of the tunnel face generates a great threat to the safety of existing pipeline.

**Fig. 11:**
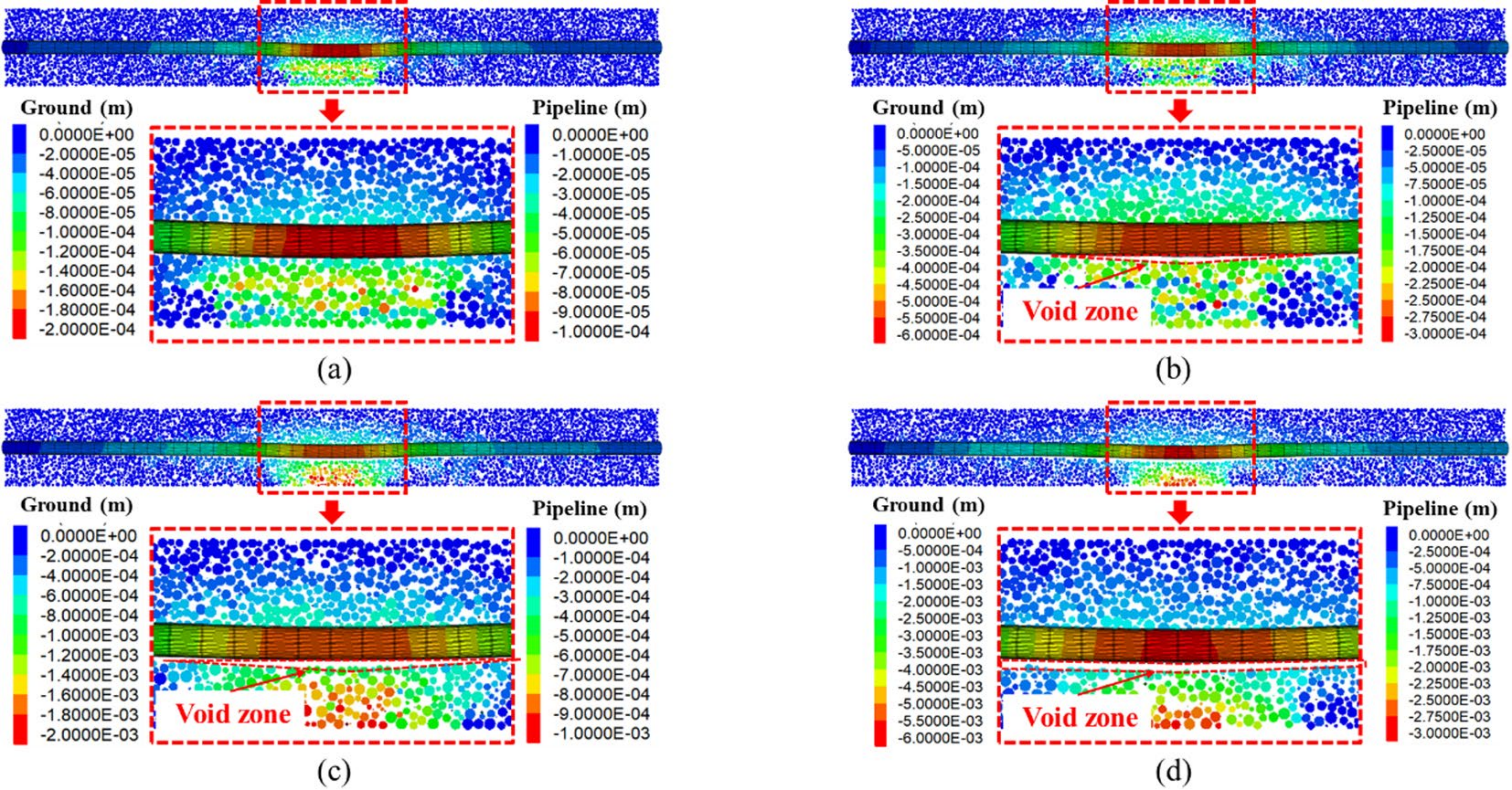
The development of soil-pipeline interaction with the movement of tunnel face. (**a**) *δ*/*D* = 0.86%, (**b**) *δ*/*D* = 3.48%, (**c**) *δ*/*D* = 5.65%, (**d**) *δ*/*D* = 6.52%.

Figure 11 shows the development of soil-pipeline interaction induced by tunnel face instability, where a void zone can be obviously observed with tunnel face movement. The void zone indicates that discontinuous contact occurs between ground and pipeline, the mechanisms can be explained as follows: the instability of the tunnel face causes a large amount of ground loss under the existing pipeline, and the surrounding soil cannot be filled in a timely manner. Moreover, due to the difference in stiffness between the existing pipeline and the ground, the deformation of the ground cannot be coordinated with the existing pipeline, resulting in a void zone below the existing pipeline.

**Fig. 12:**
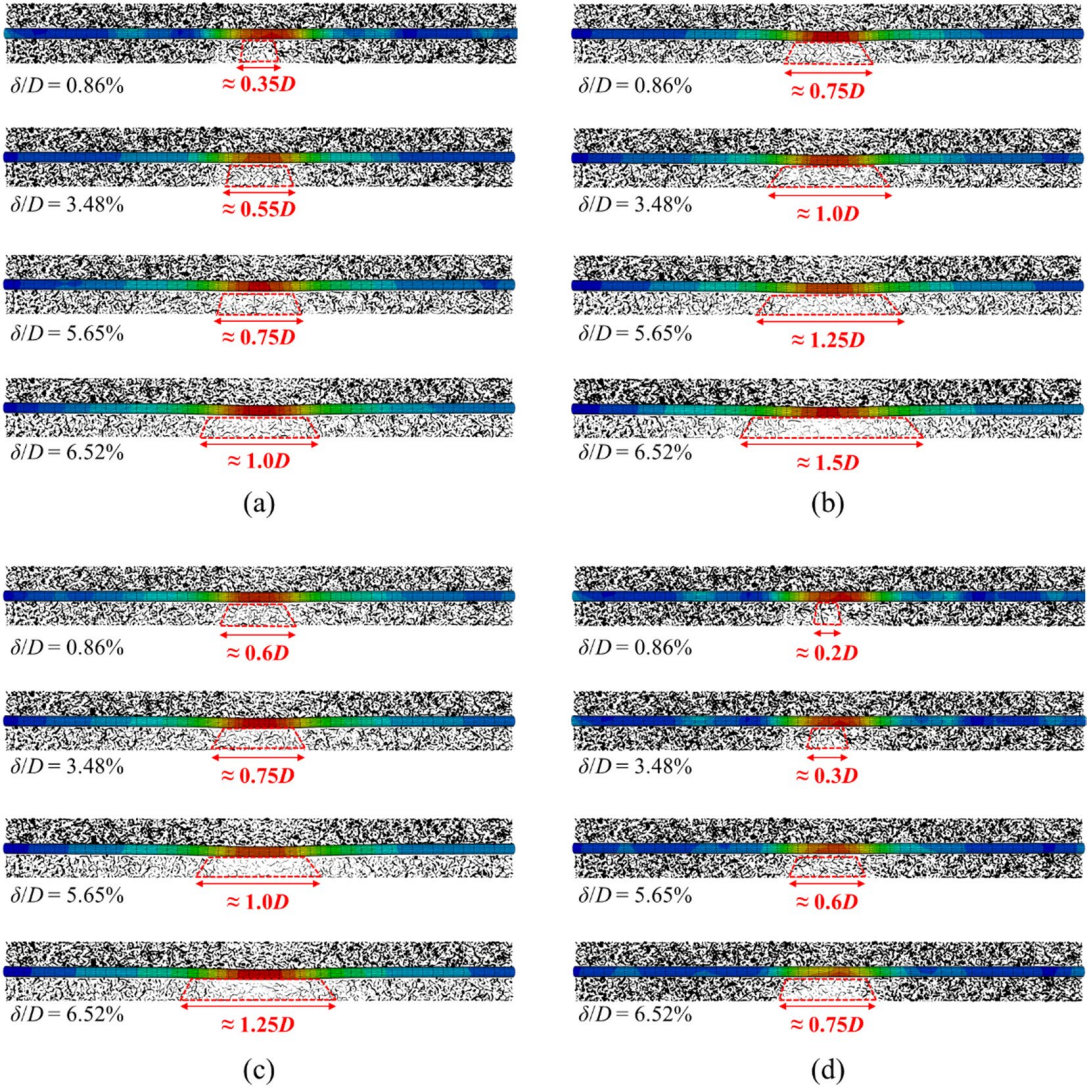
The development of contact force chain of soil particles with the movement of tunnel face. (**a**) *h*/*D* = 0.5, (**b**) *h*/*D* = 0.3, (**c**) *h*/*D* = 0.1, (**d**) *h*/*D* = 0.

Through the contact force chain of the soil particles shown in Fig. 12, it can be seen that the contact force chains significantly decrease in the area below the existing pipeline, and the influence range gradually increases with the movement of tunnel face. Comparing the distribution of contact force chains around pipeline under different *h*/*D* conditions reveals that at *h*/*D* = 0.3, the contact force chains beneath the pipeline exhibit the sparsest configuration under the same tunnel face movement. This indicates the most pronounced sheltering effect of the existing pipeline, with its influence range progressively expanding from 0.75 to 1.5*D*. At other *h*/*D* values, the influence range also gradually increases with tunnel face displacement but to a lesser extent. Notably, at *h*/*D* = 0, the maximum influence range is merely 0.75*D*, demonstrating substantially reduced impact of shield tunnel excavation on the existing pipeline.

**Fig. 13:**
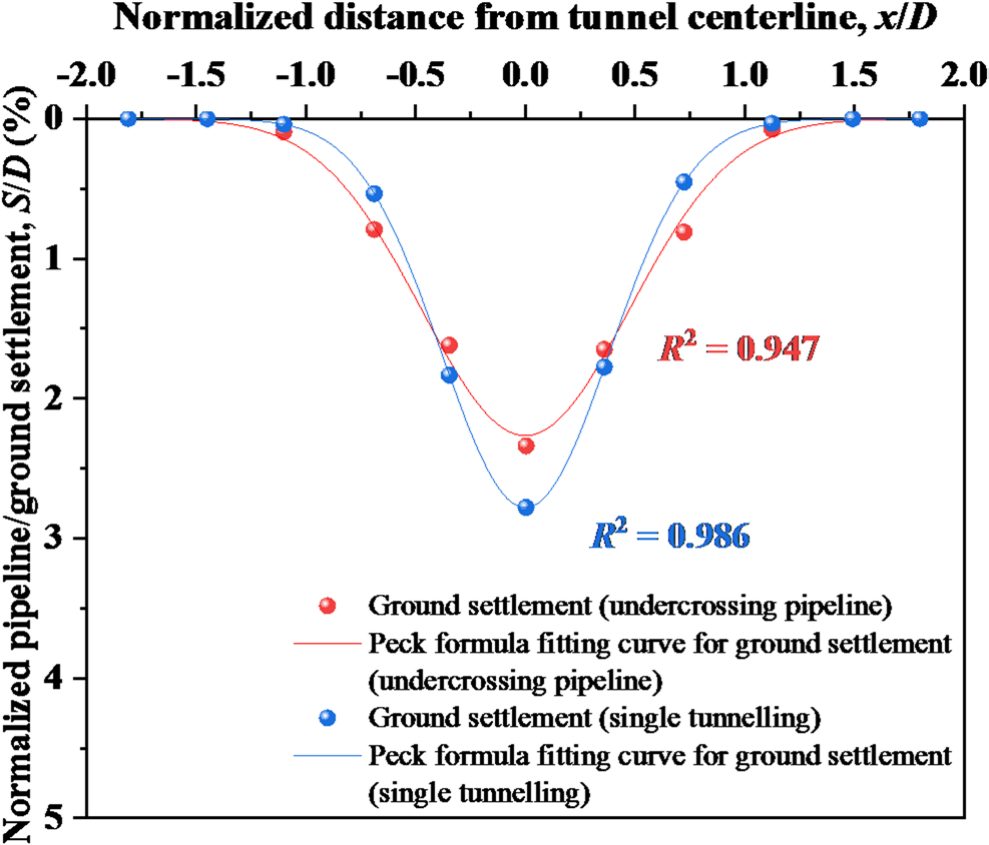
Ground settlement induced by shield undercrossing pipeline and single tunnelling.

In order to further clarify the soil-pipeline discontinuous contact mechanisms, the ground settlement induced by tunnel face instability undercrossing existing pipeline was compared with that induced by single tunnelling (i.e., greenfield settlement) under the same condition. The results are shown in Fig. 13, it can be observed that the ground settlement with the existing pipeline is smaller than greenfield settlement, which is due to the fact that the stress release below the existing pipeline causes rebound deformation, while the existing pipeline bears the soil pressure above, making it unable to act on the underlying ground. The surface settlement trough induced by tunnel face instability undercrossing existing pipeline is wider than that induced by single tunnelling, demonstrating that existing pipeline redirect soil deformation toward broader zones. Furthermore, surface settlement with existing pipeline exhibits reduced magnitude directly above the tunnel centerline compared to greenfield settlement, but exceeds it laterally on both sides. This pattern indicates soil-pipeline separation directly above the tunnel centerline and compression on both sides, suggesting that the soil-pipeline interaction analyses must account for differential contact states.

**Fig. 14:**
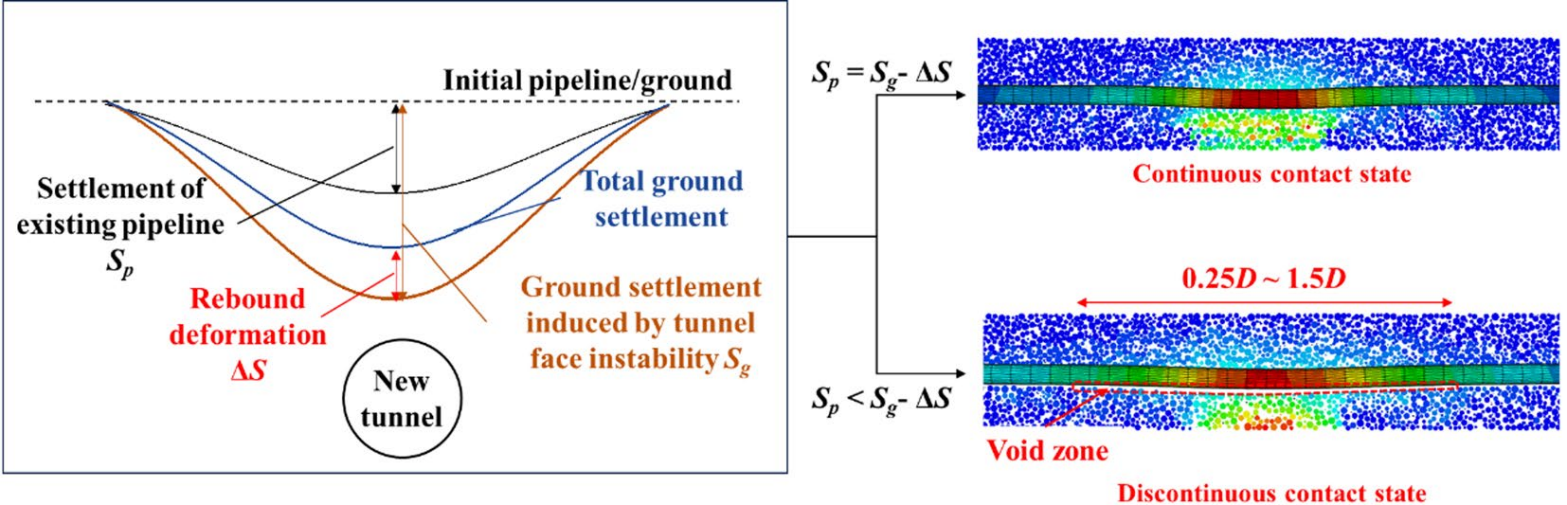
Soil-pipeline discontinuous contact mechanisms induced by tunnel face instability.

Based on the above findings, it can be concluded that the soil-pipeline discontinuous contact mechanisms induced by the instability of shield tunnel face can be described as follows: the instability of tunnel face causes soil movement, leading to the settlement deformation and the rebound deformation in the ground simultaneously. If the sum of the settlement deformation and the rebound deformation (i.e. the total deformation of the ground) is consistent with the deformation of the existing pipeline, they will maintain the continuous contact state. However, once the total deformation of the ground is greater than that of the existing pipeline, a void zone will appear, resulting in discontinuous contact between the ground and existing pipeline. In such circumstances, the ground beneath the existing pipeline will exhibited separation directly above the tunnel centerline and compression on both sides, respectively. From this study, the separation zone ranges from 0.25 to 1.5*D* with the movement of tunnel face. The schematic diagram of the soil-pipeline interaction as mentioned above is shown in Fig. 14.

## Discussions

### Effect of vertical spacing on pipeline settlement

**Fig. 15:**
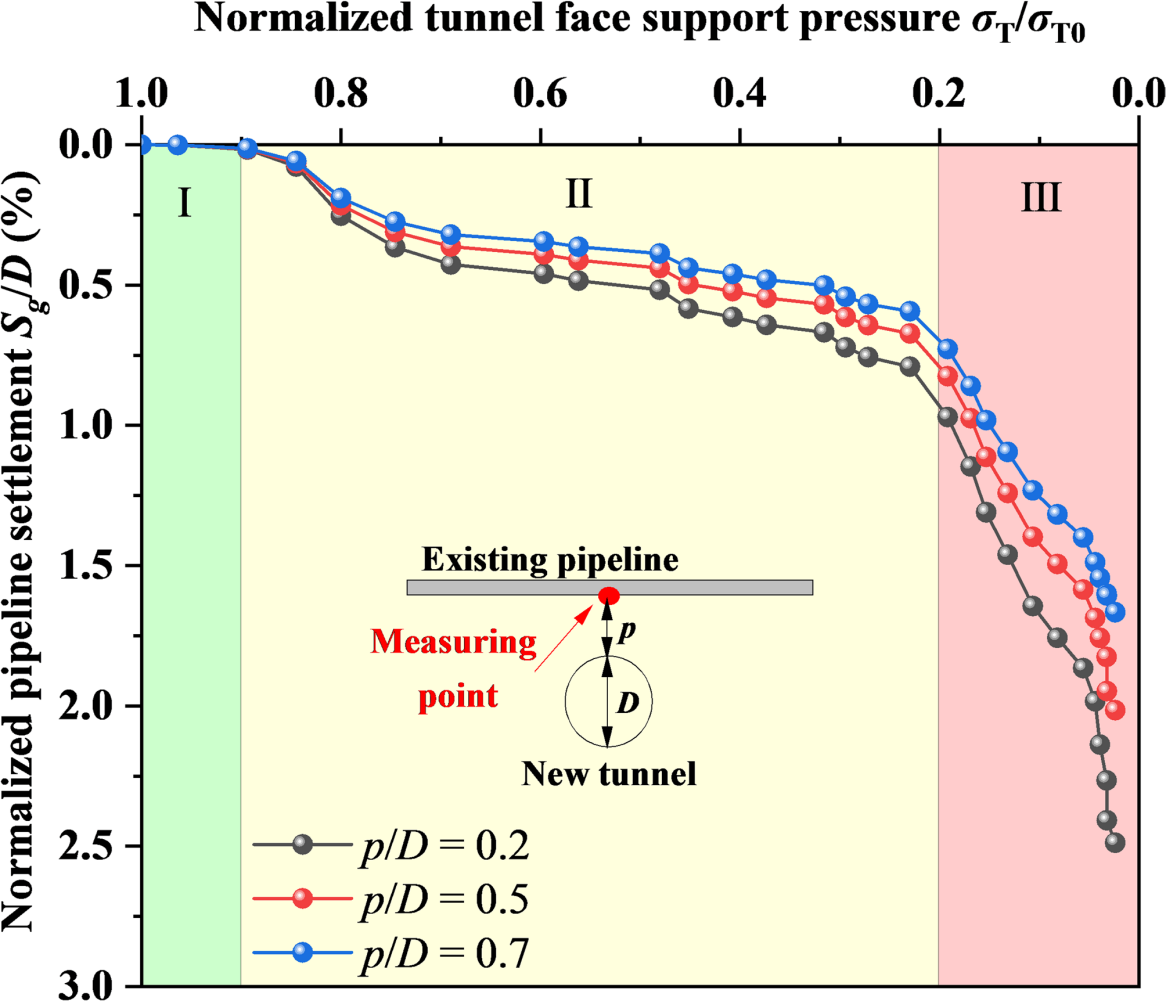
The relationship between tunnel face support pressure and the pipeline settlement (measuring point locates directly above the new tunnel).

The average contact stress of soil particles on the shield tunnel face was recorded as the face support pressure, and the relationship between the face support pressure and the maximum deformation at the center section of the existing pipeline at different spacings was obtained at *h*/*D* = 0.3, as shown in Fig. 15. It can be seen that as the face support pressure decreases, the deformation development at the center of the existing pipeline presents a three-stage characteristic: The first stage is when the face support pressure is between *σ*_T0_ and 0.9*σ*_T0_ (where *σ*_T0_ indicates the initial tunnel face support pressure), and the existing pipeline hardly deforms due to its high stiffness, indicating that this stage is a safe stage for the existing pipeline; The second stage is when the face support pressure is between 0.9*σ*_T0_ and 0.2*σ*_T0_. At this time, the existing pipeline gradually deforms. Although the overall trend is relatively flat, when the support pressure drops to 0.2*σ*_T0_, the deformation has reached more than 0.7%*D*, corresponding to 50 mm in prototype, which greatly exceeds the safety deformation range of the existing pipeline; The third stage is when the face support pressure is reduced to below 0.2*σ*_T0_. At this time, the deformation of the existing pipeline increases sharply. This is due to severe instability of the tunnel face and ground loss, causing the existing pipeline to be in a dangerous state. The pressure of the upper ground and the self-weight of the existing pipeline concentrate on the pipeline structure, and there is no support from the underlying ground, resulting in serious deformation of the existing pipeline and threatening its structural safety. Given that the pipeline’s sheltering effect on soil displacement is most significant at *h*/*D* = 0.3 (as shown in Fig. 7b), implying the pipeline bears the maximum earth pressure and is at its least safe condition, the support pressure threshold obtained under this condition is inherently conservative.

In addition, with the increase of vertical spacing, the deformation of existing pipeline slightly decreases, which is due to that the larger the vertical spacing, the lower the disturbance transmission effect. Although increasing the vertical spacing can reduce the deformation of existing pipeline to a certain value, the effect is limited. It is still necessary to control the face support pressure above 0.9*σ*_T0_ in order to better ensure the safety of existing pipeline.

### Effect of flexural stiffness on pipeline settlement

**Fig. 16:**
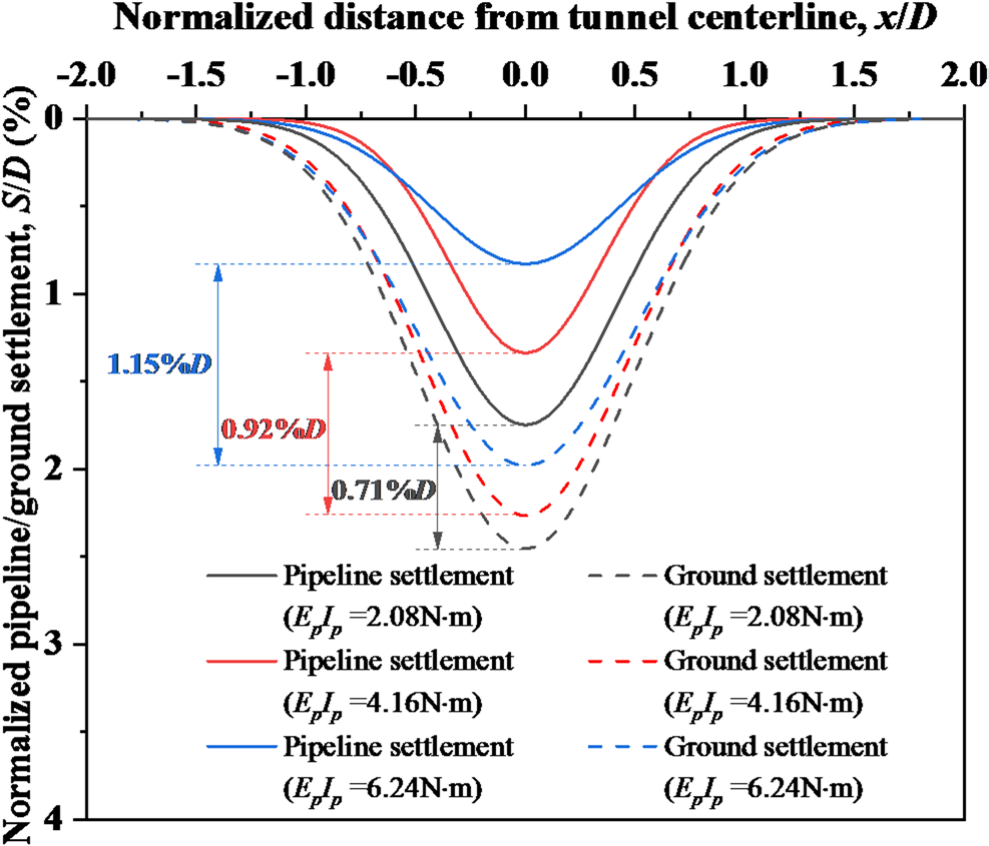
Settlement of ground and existing pipeline under varying flexural stiffness of pipeline.

Shield undercrossing inevitably induces ground deformation and consequent pipeline deformation, where pipeline flexural stiffness directly governs the deformation. Figure 16 compares pipeline settlements and ground settlements under varying flexural stiffness during tunnel face instability, revealing that increased flexural stiffness significantly reduces maximum pipeline deformation. Concurrently, pipeline with lower flexural stiffness exhibits wider settlement trough due to enhanced compliance with ground settlement. Furthermore, quantitative analysis of the height of void zone (differential settlement between pipeline and ground) demonstrates growth from 0.71%*D* (1.63 mm) to 1.15%*D* (2.65 mm) as flexural stiffness *E*_*p*_*I*_*p*_ rises from 2.08 to 6.24 N·m. This progression signifies intensified discontinuous pipe-soil interaction, substantially compromising pipeline safety.

**Fig. 17:**
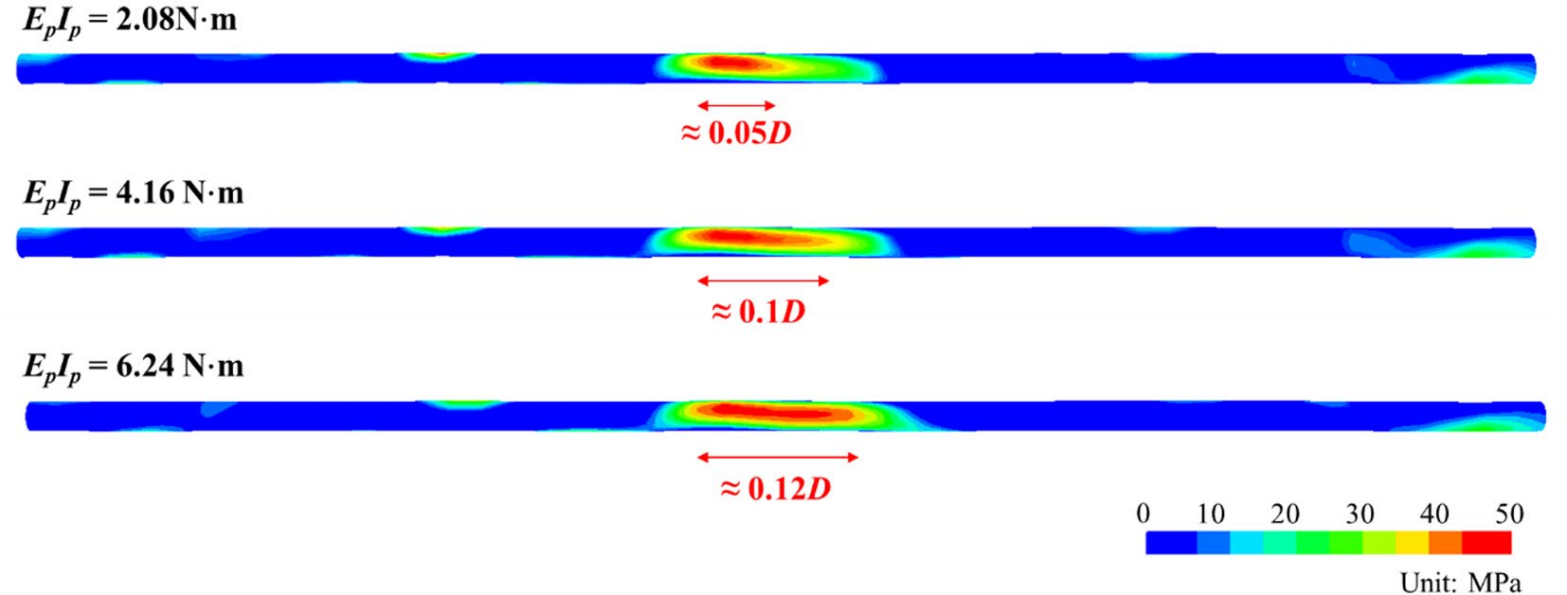
Longitudinal tensile stress distribution along the pipeline bottom under varying flexural stiffness of pipeline.

To better assess damage risk, stress and strength analyses of the pipeline under varying stiffness conditions were conducted. The pipeline material is PVC with a typical tensile strength of 50 MPa under bending moment. Figure 17 presents the longitudinal tensile stress distribution along the pipeline bottom. As pipeline stiffness *E*_*p*_*I*_*p*_ rises from 2.08 to 6.24 N·m, the failure zone (red areas where stress exceeds 50 MPa) expands from 0.25 to 0.4*D*. This occurs because increased stiffness enlarges void zone, forcing the pipeline to bear greater overlying earth pressure. Thus, while higher pipeline stiffness controls deformation, it significantly increases both void extent and stress magnitude, indicating that practical engineering requires optimal stiffness selection—avoiding excessive or insufficient values.

### Effect of tunnelling speed on pipeline settlement

Tunnelling speed critically modulates soil-structure interactions and failure mechanisms due to its substantial impact on surrounding ground, which governs face stability and pipeline behavior. Previous studies^62,63^ demonstrate that higher tunnelling speed reduces surrounding ground displacement and stress release, resulting in higher support pressure. Within moderate speed variations, support pressure approximates a linear relationship with tunnelling speed. For comparative analysis, normalized parameters were employed:

2 $$\frac{{v_{T} - v_{T0} }}{{v_{T0} }} = a\frac{{\sigma_{T} - \sigma_{T0} }}{{\sigma_{T0} }}$$

where *v*_T_ and *v*_T0_ are the changed and the initial tunnelling speed, respectively; *σ*_T_ and *σ*_T0_ are the changed and the initial support pressure, respectively.

**Fig. 18:**
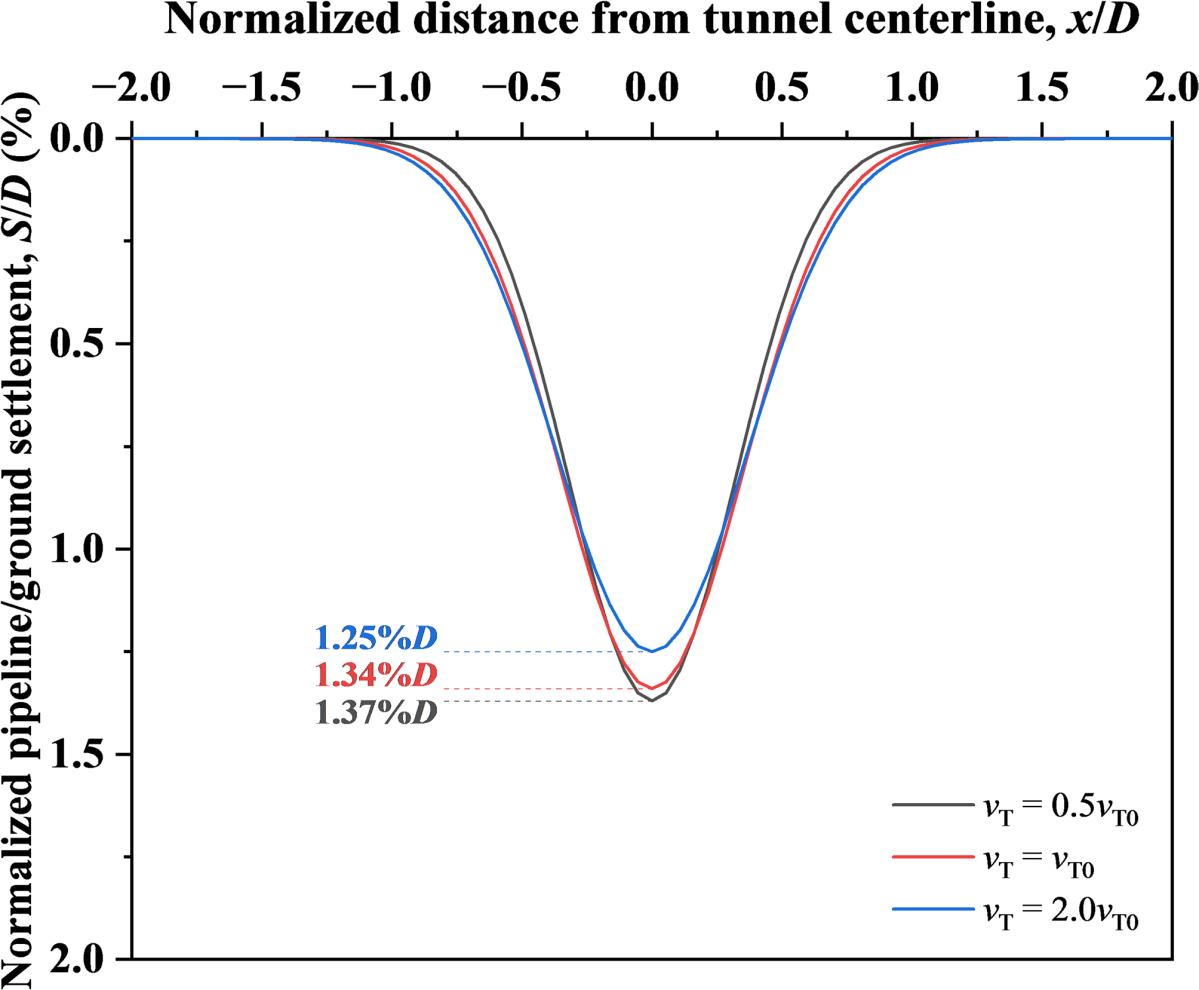
Settlement of ground and existing pipeline under varying tunnelling speed.

As illustrated in the literature^62^, when tunnelling speed increases from 5 to 20 m/d, maximum support pressure rises from 3.4 to 3.7 MPa, yielding the coefficient *a* = 34. Simulations considered speeds of 0.5*v*_T0_ and 2.0*v*_T0_, corresponding to normalized support pressures of 0.985*σ*_T0_ and 1.029*σ*_T0_ via Eq. (2). Subsequently, the support face was pre-displaced to these pressures before DCM implementation. Figure 18 demonstrates reduced pipeline settlement (from 1.37 to 1.25%*D*) with increased tunnelling speed (0.5–2.0*v*_T0_), which is attributable to heightened support pressure enhancing soil densification and mitigating instability impacts.

## Conclusions

In this paper, three-dimensional finite-discrete numerical simulations were applied to investigate the response of ground and existing pipeline to face instability of an undercrossing shield tunnel. Based on the numerical results, the ground response including failure modes, microscopic mechanism, and ground settlement were thoroughly studied, and the soil-pipeline discontinuous contact mechanisms were further analyzed. It should be emphasized that the mechanisms and quantitative conclusions derived in this study are based on sandy soil conditions, which are rooted in the frictional and stress-redistribution behavior of sandy soil. The applicability to clayey soil requires further investigation based on the characteristics of clay-pipeline interactions. The main conclusions are as follows:

1. Ground failure modes predominantly exhibit wedge-shaped configurations, with pipeline demonstrating a sheltering effect on ground movements. As the tunnel face advances toward existing pipeline (*h*/*D* decreasing from 0.5 to 0.1), the sheltering-affected zone transitions from an inverted triangular zone to a spindle-shaped zone. At *h*/*D* = 0, the sheltering effect essentially dissipates.
2. The evolution of contact force chain during tunnel face instability reveals that soil particle slippage and migration constitutes the microscopic mechanism of instability. Existing pipeline interrupts force chain transmission paths, resulting in sparser contact force chain beneath pipelines which induce additional pipeline stresses and deformations.
3. Ground surface settlements induced by face instability form dual troughs on both sides of pipeline, exceeding 3%*D* (6.9 mm). Soil-pipeline discontinuous contact mechanisms originate from differential deformation between ground and pipeline, manifested as void zones beneath pipelines, As the tunnel face gradually becomes unstable, the degree of discontinuity increases from 0.25 to 1.5*D*. When tunnel face movement *δ*/*D* increases from 0.86 to 6.52%, the contact between ground and pipeline transitions from continuous state to discontinuous state with maximum void height reaching 2.3 mm, while the stress loosening zone expands from 0.75 to 1.5*D*.
4. The pipeline deformation progresses through three stages with decreasing face support pressure: deformation stabilizes when *σ*_T_ > 0.9*σ*_T0_, gradually increases when 0.2*σ*_T0_ < *σ*_T_ < 0.9*σ*_T0_, and accelerates rapidly when *σ*_T_ < 0.2*σ*_T0_. Parametric analysis shows vertical spacing exerts limited influence on pipeline settlement, whereas increased flexural stiffness from 2.08 to 6.24 N·m causes void height growth from 0.71 to 1.15%*D* and failure zone from 0.25 to 0.4*D*.

## Data Availability

No datasets were generated or analysed during the current study.
